# The Walker circulation shaped by tectonics

**DOI:** 10.1093/nsr/nwaa133

**Published:** 2020-06-18

**Authors:** Yongyun Hu

**Affiliations:** Department of Atmospheric and Oceanic Sciences, School of Physics, Peking University, China

The Walker circulation is a thermally driven tropical atmospheric circulation in the longitudinal direction. At present, the ascending branch of the Walker circulation is over the warm pool in the tropical western Pacific and the descending branch is over the tropical eastern Pacific where sea-surface temperatures (SSTs) are cooler. How could the Walker circulation be a few tens of million years ago (Ma)? How did it evolve over tectonic time scales?

Yan *et al.* [1] demonstrated simulations that the Walker circulation has been narrowed by ∼38° in longitude since the Early Cenozoic and that the strength of the Walker circulation increased with time until the Late Miocene, then started decreasing. They concluded that the changes in both width and strength are mainly due to changes in continental distributions and that CO_2_-concentration changes also play an important role in causing the strength change.

**Figure 1.**
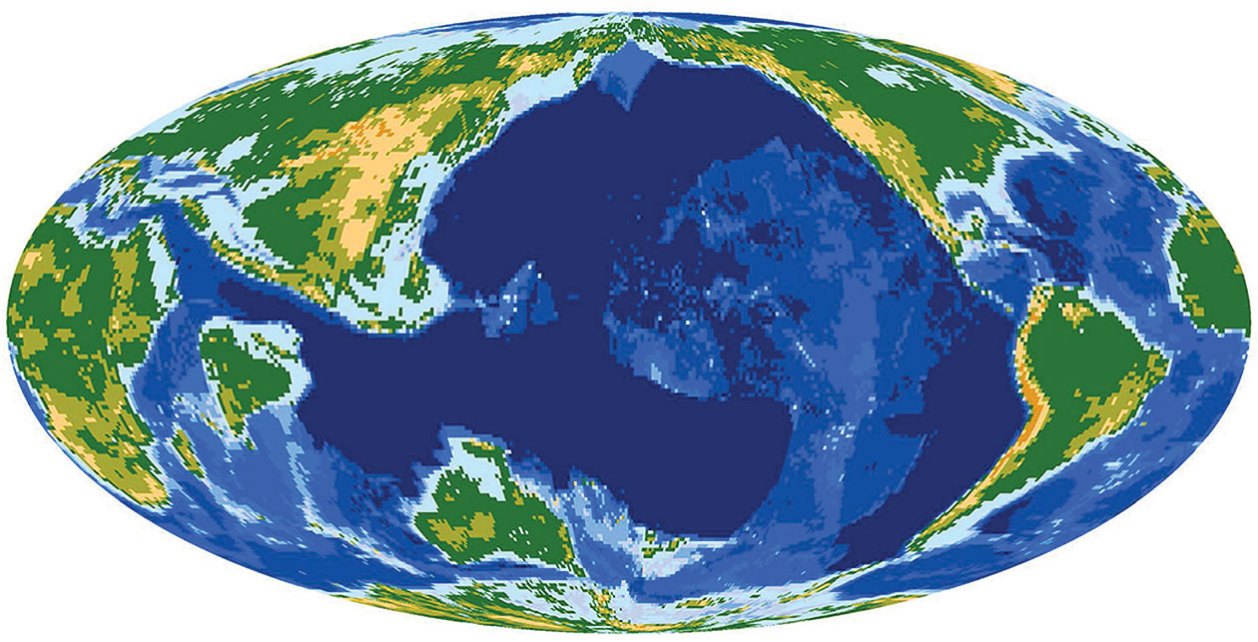
**Figure 1.** Continent distribution at 60 Ma. Produced based on data from Scotese [2].

There are two major differences in climate conditions between the Early Cenozoic and the present: continental distributions and atmospheric CO_2_ concentrations. Both are associated with the evolution of plate tectonics. Figure 1 illustrates the continental distribution at 60 Ma [2]. The Indian continent had just reached the equator as it was moving northeastward, the Australian continent had just broken away from the Antarctic continent, the South American continent was still far to the east relative to its present location, and the North and South American continents remained well separated. Such a continental distribution resulted in a much broader ocean basin compared with that at present; especially, the tropical Pacific and Indian Oceans were well connected. As a result, the warm

pool was far to the west relative to the present location, the cool pool was far to the east and the Walker circulation was much wider in the Early Cenozoic.

By the Late Pliocene, the Indonesian and Panama seaways were all closed because of northward moving of the Australian and South American continents, resulting in a much narrower ocean basin in the Pacific. The warm and cool pools was pushed closer, causing the narrower Walker circulation at present.

Plate-tectonics evolution impacted on not only the width, but also the strength, of the Walker circulation. Yan *et al.* [1] showed that the strengthening of the Walker circulation between the Early Cenozoic and the Late Miocene is due to systematic decrease of CO_2_ concentrations. This is consistent with previous simulations that a CO_2_ decrease would cause a sharper SST contrast between the western and eastern tropical Pacific and hence a stronger Walker circulation [3]. They also demonstrated that the closure of the Panama seaway caused weakening of the Walker circulation since the Late Miocene.

The results by Yan *et al.* [1] have important implications that tectonics can largely shape atmospheric and oceanic circulations, such as the Hadley circulation, regional monsoon circulations, El Niño and Southern Oscillation. In addition, more fundamental issues related to Yan *et al.* [1] are how biogeochemical processes associated with plate tectonics caused the systematic CO_2_ decrease since the Early Cenozoic and how the coupling between the fluid and solid spheres led to the systematic global cooling. These questions remain great challenges to Earth sciences.

***Conflict of interest statement***. None declared.
